# Early-life nicotine or cotinine exposure produces long-lasting sleep alterations and downregulation of hippocampal corticosteroid receptors in adult mice

**DOI:** 10.1038/s41598-021-03468-5

**Published:** 2021-12-13

**Authors:** Stefano Bastianini, Viviana Lo Martire, Sara Alvente, Chiara Berteotti, Gabriele Matteoli, Laura Rullo, Serena Stamatakos, Alessandro Silvani, Sanzio Candeletti, Patrizia Romualdi, Gary Cohen, Giovanna Zoccoli

**Affiliations:** 1 PRISM Lab, Department of Biomedical and Neuromotor Sciences, Alma Mater Studiorum, University of Bologna, Piazza di Porta San Donato 2, 40126 Bologna, Italy; 2 Department of Pharmacy and Biotechnology, Alma Mater Studiorum, University of Bologna, Bologna, Italy; 3 Department of Women and Child Health, Karolinska Institutet, Stockholm, Sweden; 4 Centre for Sleep Health and Research, Sleep Investigation Laboratory, Royal North Shore Hospital, Sydney, Australia

**Keywords:** Neurophysiology, Circadian rhythms and sleep, Epigenetics in the nervous system, Stress and resilience

## Abstract

Early-life exposure to environmental toxins like tobacco can permanently re-program body structure and function. Here, we investigated the long-term effects on mouse adult sleep phenotype exerted by early-life exposure to nicotine or to its principal metabolite, cotinine. Moreover, we investigated whether these effects occurred together with a reprogramming of the activity of the hippocampus, a key structure to coordinate the hormonal stress response. Adult male mice born from dams subjected to nicotine (NIC), cotinine (COT) or vehicle (CTRL) treatment in drinking water were implanted with electrodes for sleep recordings. NIC and COT mice spent significantly more time awake than CTRL mice at the transition between the rest (light) and the activity (dark) period. NIC and COT mice showed hippocampal glucocorticoid receptor (GR) downregulation compared to CTRL mice, and NIC mice also showed hippocampal mineralocorticoid receptor downregulation. Hippocampal GR expression significantly and inversely correlated with the amount of wakefulness at the light-to-dark transition, while no changes in DNA methylation were found. We demonstrated that early-life exposure to nicotine (and cotinine) concomitantly entails long-lasting reprogramming of hippocampal activity and sleep phenotype suggesting that the adult sleep phenotype may be modulated by events that occurred during that critical period of life.

## Introduction

Stressors acting during critical windows of development may lead to permanent changes in the fetus, which may promote early-life survival while predisposing the individual to disease later in life^1^. These stressors include malnutrition, depression, lack of maternal behavior, as well as the exposure to substances of abuse such as opioids, ethanol or nicotine^1–3^. Several studies have shown the negative impact of perinatal stress on neurogenesis^4^, mood^5^, metabolism^3^, and cardiovascular regulation^6,7^ in adult rodents. On the other hand, a clearly established relationship between perinatal stress and widely prevalent sleep alterations or disorders, such as sleep fragmentation or insomnia, respectively^8^, has been hypothesized but is still missing^2^.

Plasma cortisol levels physiologically increase during pregnancy especially in the last trimester of gestation, but the amount that reaches the fetus is carefully controlled by the placental glucocorticoid-inactivating enzyme 11β-HSD2^9^. However, maternal stress may downregulate this placental enzyme^10^ overexposing the fetus to glucocorticoids, which may impact on the correct development and responsiveness of the hormonal stress response throughout life. In particular, the hippocampus is considered the most stress-sensitive structure of the developing brain because it is a highly plastic region^1^, it mostly develops postnatally^11,12^, and it is rich in corticosteroid receptors^13^. The hippocampus physiologically exerts an inhibitory regulation of the hypothalamic–pituitary–adrenal (HPA) axis^13^ by reducing the hypothalamic release of the corticotropin releasing factor (CRH) and, consequently, of circulating cortisol in humans or of corticosterone in rodents. This inhibitory action is due to the negative feedback regulation exerted by the continuous monitoring of blood cortisol by the 2 hippocampal corticosteroid receptors: the mineralocorticoid and the glucocorticoid receptors (MRs and GRs, respectively)^14^.

Electrophysiological and behavioral studies suggest that GR and MR may exert different, and even functionally antagonistic, effects^15^. For instance, in the hippocampus, MR activation maintains excitability and regulates behavioral reactivity and response selection, while GR occupancy suppresses excitability and facilitates storage of information^15^. On the other hand, MR and GR actions on HPA axis regulation appear to be synergistic^14^. Hippocampal MR is important in controlling the basal inhibitory tone along the HPA axis and at the onset of the stress response. This effect of cortisol via MR is modulated by GR, which facilitates the termination of the physiological stress response^16^. The role of GR in the stress response has been widely investigated. Several experiments agree with the notion that perinatal stress epigenetically downregulates hippocampal GR expression in newborns, thus hyperactivating their HPA axis^1,17^. On the contrary, the role of MR in this picture is still undefined since contradictory results have been produced^18–20^. Based on these considerations, the effects of stress and corticosteroids in the hippocampus may be evaluated in terms of MR/GR balance rather than considering these receptors separately. This approach has been proved to work in Post-Traumatic Stress Disorder models^20,21^, but it has never been assessed in the relationship between perinatal stress and adult sleep phenotype.

Perinatal exposure to nicotine has been related to hyperactivation of the stress response in infant humans^22^ and adult rats^23^. Therefore, maternal smoking may be rightfully considered an early-life stressor, which is still widely diffused in both industrialized countries^24^ and indigenous populations^25^. Nicotine is the major active molecule of tobacco^26,27^. However, tobacco contains many different compounds and nicotine itself is metabolized in more than 20 different molecules^26^. Cotinine, the main product of nicotine metabolism, is structurally very similar to nicotine but it has a different mode of action: it is a positive allosteric modulator of acetylcholine receptors^28^. Cotinine thus potentiates the responsiveness of acetylcholine receptors to natural ligands, such as acetylcholine and choline, and to agonists such as nicotine^28^. Moreover, cotinine has longer half-life (> tenfold)^26^ than nicotine and it can bind to at least one independent receptor^29^. Altogether, the available evidence suggests considering cotinine a potentially harmful molecule as well as nicotine and not just its inert product^30^. The use of nicotine replacement therapy is a highly controversial strategy to help pregnant smokers to quit smoking. Even though this strategy is less harmful than continuing smoking^31^ it must be kept in mind that both nicotine and cotinine cross the placental barrier and easily pass from blood to milk^7,32^. It has been postulated that nicotine from maternal smoking may impact on the developing brain’s acetylcholine neurotransmitter systems and neurons involved in sleep regulation^33–36^. However, convincing evidence on persistent long-term effects of this modulation is lacking.

With the present study, we wanted to investigate the long-term association between maternal nicotine and cotinine exposure, hippocampal MR and GR expression, and adult sleep. Independent experiments have already shown that maternal nicotine exposure produces downregulation of placental 11β-HSD2, exposing fetuses to high corticosteroid levels and impacting on their steroidogenesis and HPA axis development^37,38^. Early-life nicotine exposure produces persistent sleep alterations in humans, with increased parasomnias in adolescents^34^, and in adult mice, with decreased wakefulness and altered sleep homeostasis^33^. Early-life nicotine exposure also downregulates hippocampal GR and MR expression in rodents^23^. However, the extent to which these effects are due to cotinine, which is nicotine’s main metabolite, remains unclear because none of these effects has been investigated on subjects perinatally exposed to cotinine.

Considering this evidence, we aimed at investigating whether the perinatal exposure to either nicotine or its principal metabolite, cotinine, entails long-lasting reprogramming of the wake-sleep pattern and of the hippocampal corticosteroid receptor expression in adult mice. We also aimed to explore the epigenetic mechanisms which might explain this long-lasting reprogramming.

## Methods

### Ethical approval

The study protocol complied with the EU Directive 2010/63/EU for animal experiments and was approved by the Committee on the Ethics of Animal Experiments of the University of Bologna (Prot. n. CES60 All:14, 27/06/2013) and of the Italian Ministry of Health (Prot. n.170/2011-B). The experiments were carried out according to the guidelines of the animal welfare committee of the University of Bologna, Italy, and ARRIVE guidelines. All efforts were made to minimize animal suffering.

### Mice and drugs

**Figure 1:**
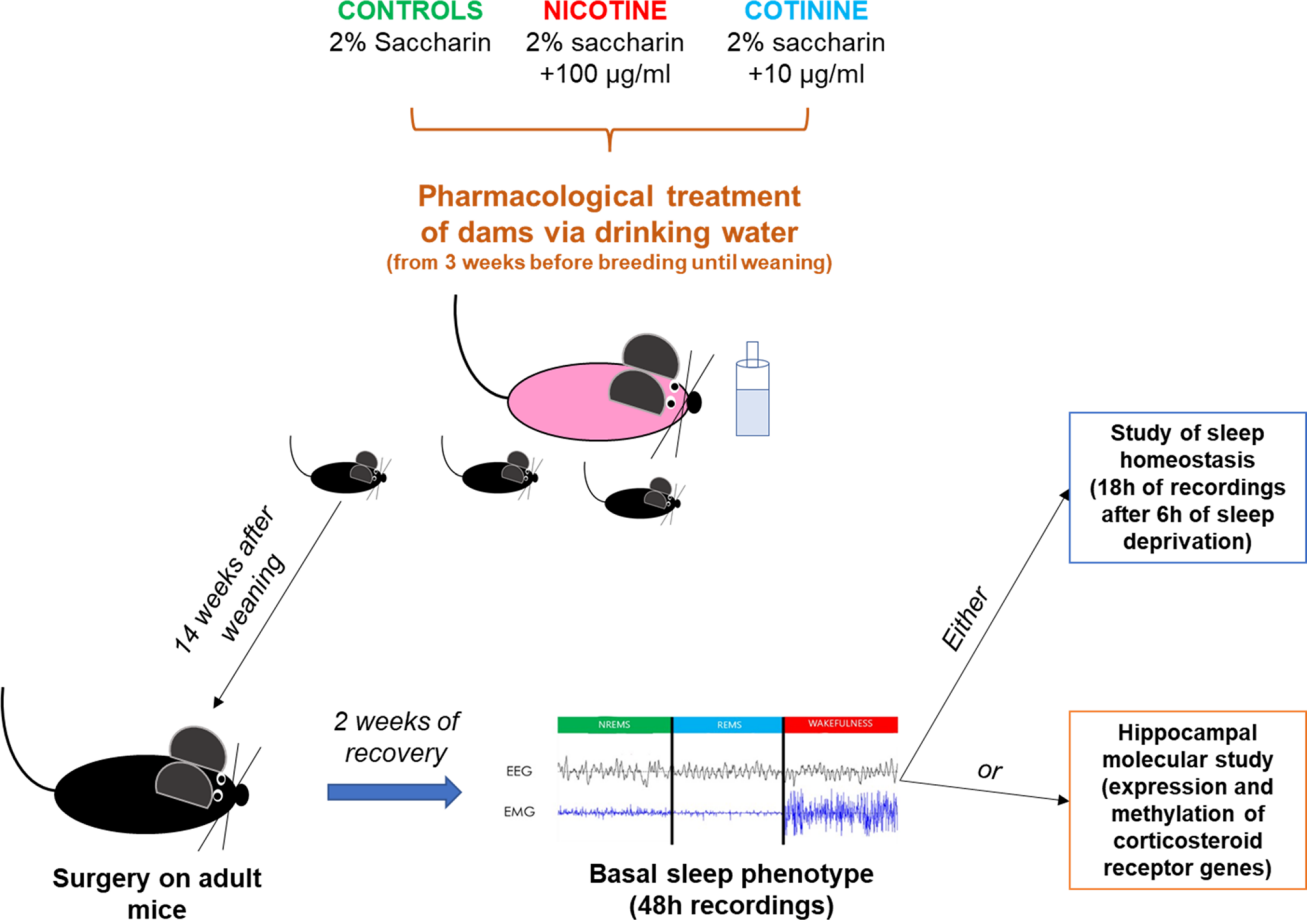
Graphical representation of experimental design. The picture shows the scheme of the experimental protocol used in this study (cf. Methods). C57Bl/6 J dams (3–4/cage) drank water laced with 2% saccharin, or 2% saccharin + 100 µg/mL nicotine freebase or 2% saccharin + 10 µg/mL cotinine for 3 weeks prior to and 6 weeks after mating. Pups never drank these solutions. Adult (≈ 17 weeks old) male mice underwent surgery to implant electrodes for electroencephalographic (EEG) and electromyographic (EMG) signals. After at least 2 weeks of recovery, basal sleep phenotype was assessed by recording EEG/EMG signals for 48 h. After that, mice were split in 2 subgroups to evaluate either the sleep homeostasis (after 6 h of sleep deprivation) or the hippocampal expression and methylation of corticosteroid receptor genes.

Mouse breeding was performed with harem matings with 3–4 C57Bl/6J dams per cage. Mice were housed in standard cages with floor area of 530 or 820 cm^2^ (Cat. No 1284L and 1290D by Tecniplast, Varese, Italy) also depending on the number of females and with specific wood particles as bedding (Scobis One by Mucedola, Milano, Italy). With this breeding scheme, usually, 1 or 2 pregnancies occurred at the same time. In this case, we kept the harem as it was until pups were weaned. However, when occasionally more than 2 pregnancies occurred at the same time, dams were separated before delivery to prevent crowding and to correctly keep track of the pups’ age. Dams (3–4/cage) drank water laced with 2% saccharin, or 2% saccharin + 100 µg/mL nicotine freebase (Sigma Aldrich, N6763) or 2% saccharin + 10 µg/mL cotinine (Sigma Aldrich, C5923) for 3 weeks prior to and for 6 weeks after mating. Saccharin (a sweetener) was added to drinking water to mask the bitter flavor of nicotine and cotinine. Drug-laced drinking water was refreshed twice weekly, and we concomitantly monitored fluid intake in a subset of cages. Pups never drank these solutions (Fig. 1). We did not keep records about the exact number of litters or of mice per litter included in the study, however, we did not need to manually equalize the litter size since litters were comparable between experimental groups in terms of number of pups and weight at birth^7^.

We previously showed that these doses of cotinine and nicotine produced similar plasma cotinine levels both in dams and their pups^7^ and that these concentrations were comparable to those detected in the blood of human newborns of passive smokers^39^. The fact that different doses of cotinine and nicotine produced similar plasma cotinine levels has already been observed in acute experiments on mice^40^ and might be linked to the slow release of nicotine from tissue to blood, which extends the half-life of cotinine derived from nicotine beyond that of directly administered cotinine^41^. After weaning, pups were kept under a 12:12-h light–dark cycle at 25 °C with free access to drug-free drinking water and food (4RF21 diet; Mucedola, Milano, Italy) and with lights on (Zeitgeber Time 0, ZT0) at 9 am.

### Experimental protocol

Only adult male mice born from nicotine- (NIC, *n* = 15), cotinine- (COT, *n* = 14) or just saccharin- (control group, CTRL, *n* = 12) treated dams were included in the present experiment. Data on cardiovascular regulation of 23 mice (9 NIC mice, 8 COT mice, and 6 CTRL mice) out of 41 mice included in this experiment were previously published for different purposes^7^. A new batch of mice (6 NIC mice, 6 COT mice, and 6 CTRL mice) studied with the same drug administration and surgery protocol (Fig. 1) was added to the present study with the purpose of increasing sample size and robustness of results on wake-sleep states, hippocampal gene expression and DNA methylation (see below).

Adult male mice underwent surgery at 18.3 ± 0.4 weeks of age with no significant difference between experimental groups. At surgery, COT mice were significantly (cf. Statistical Appendix) heavier than CTRL mice (29.2 ± 0.5 vs. 27.0 ± 0.4 g), whereas NIC mice were not (27.5 ± 0.6 g). Each adult mouse was anesthetized and injected with analgesic (1.8–2.4% isoflurane in O_2_ + Carprofen 0.1 mg sc; Pfizer Italy, Latina, Italy). Mice were then implanted with a pair of Teflon-coated stainless-steel electrodes (Cooner Wire, Chatsworth, CA, USA) in contact with the dura mater through burr holes in the frontal and parietal bones to obtain a differential electroencephalographic (EEG) signal. A second pair of these electrodes was inserted bilaterally in the nuchal muscles to obtain a differential electromyographic (EMG) signal. All electrodes were connected to a miniature custom-built socket, which was cemented to the skull with stainless-steel anchor screws (2.4 mm length, Plastics One, Roanoke, VA, USA), dental cement (Rely X ARC, 3 M ESPE, Segrate, MI, Italy), and dental acrylic (Respal NF, SPD, Mulazzano, Italy). A dummy telemetric arterial blood pressure transducer (TA11-PAC10, DSI, Tilburg, Netherlands) was implanted, with the catheter tip advanced via the femoral artery into the abdominal aorta just caudal to the renal arteries. Catheter implantation was performed to keep the surgical protocol for the new batch of mice (6 NIC mice, 6 COT mice, and 6 CTRL mice) identical to that of our previously published work^7^. Blood pressure data will not be further discussed in the present study. After the surgery, mice were housed individually and allowed 7 days to fully recover. Then, mice were briefly anesthetized (1.8–2.4% isoflurane in O_2_) to connect the electrodes to a recording cable which, in turn, was plugged to a rotating swivel (SL2 + 2C/SB, Plastics One, Roanoke, VA, USA) and a balanced cable suspensor allowing unhindered movements to the mice. After 5–7 more days of habituation to the recording setup, all mice underwent baseline EEG/EMG recordings for 48 h starting at ZT0^43,44^. After these baseline recordings, the 23 mice (9 NIC mice, 8 COT mice, and 6 CTRL mice) that had been included in our previous publication^7^ and 1 more NIC mouse also underwent 6 h of sleep deprivation, starting at ZT0, followed by 18 h of sleep recovery (Fig. 1). Sleep deprivation was performed by gentle handling while monitoring real-time EEG/EMG signals. Specifically, every time the EMG tone lowered with the concomitant appearance of slow (delta: 0.5–4 Hz) EEG waves, the investigator gently tapped the edge of the cage or touched the mouse with a cotton swab to prevent it from falling asleep.

All the other mice that were not exposed to sleep deprivation were euthanized (isoflurane at 4% in O_2_) between ZT0 and ZT3 at least 2 days after the end of the baseline recordings. The hippocampi were dissected and immediately stored at −80 °C for subsequent gene expression and DNA methylation analyses (Fig. 1).

### Data acquisition and analysis

All the recordings were performed on freely behaving animals housed individually in their cages. The EEG and EMG signals were amplified and filtered (EEG: 0.3–100 Hz with 50 Hz notch filter; EMG: 100–1000 Hz with 50 Hz notch filter; 7P511J amplifiers, Grass, West Warwick, RI, USA), digitized at 16-bit and 1024 Hz, and down-sampled at 128 Hz for data storage. Data acquisition was performed by means of custom software written in Labview 8.0 (National Instruments, Austin, TX, USA, www.ni.com). Data analysis was performed with MatLab R2020a (Mathworks, Natick, MA, USA, www.mathworks.com). Wakefulness, non-rapid-eye-movement sleep (NREMS), and rapid-eye-movement sleep (REMS) were automatically scored on the basis of raw EEG and EMG signals (4-s epochs) using SCOPRISM, a validated algorithm^45^. The analysis of sleep architecture was performed with a threshold of 12 s (i.e., 3 consecutive 4-s epochs) for wake-sleep episode duration^46^. The sleep fragmentation index was calculated as the number of awakenings over the total sleep time^47^. Spectral analysis of the EEG signal was performed on artefact-free 4-s epochs using the discrete Fourier transform. Inter-individual differences in EEG spectral power were accounted for as follows. The EEG power spectrum in NREMS and REMS was expressed as a percentage of total EEG power in each state. EEG spectral power in the delta frequency range (1–4 Hz: slow-wave activity, SWA) during NREMS at baseline and during sleep recovery was expressed as a percentage of the mean SWA during NREMS at baseline between ZT0 and ZT4. To increase robustness of the estimates, SWA values were calculated on 2-h temporal bins.

In rodents, blood corticosterone level has a peak at the end of the resting (light) period and a nadir at end of the (dark) active period^48,49^. Since it has been reported that prenatal stress increased the peak but not the nadir of corticosterone level in adult rats^50^, we specifically measured the amount of time spent asleep at the transition from light to dark (LtoD) period and, as internal control, from dark to light (DtoL) period. In both cases, we calculated the sum of minutes that each mouse spent awake in the 2 h before and the 2 h after the photoperiod change (LtoD: ZT10-ZT14; DtoL: ZT22-ZT2).

### DNA and RNA extraction

Molecular analyses were conducted on 17 mice (5 NIC, 6 COT and 6 CTRL mice) recorded under baseline conditions only, without exposure to sleep deprivation. DNA and RNA were extracted by the ZR-Duet DNA/RNA MiniPrep (cat. # D7001, Zymo Research, Orange, CA, USA), which provides a quick method for the isolation of high-quality genomic DNA and total RNA. According to the manufacturer’s instructions, the frozen hippocampus was homogenized using 400 μL of DNA/RNA lysis buffer and transferred into a Zymo-Spin IIIC column to be centrifuged at 12.000×g for 1 min. Subsequently, 400 μL of ethanol were added and the solution was further transferred into a Zymo-Spin IIC column to be centrifuged again at 12.000×g for 1 min. The DNA/RNA Prep buffer was added both to the Zymo-Spin IIIC column, previously transferred into a new collection tube, and to the Zymo-Spin IIC column. After 2 washing and centrifugation steps, DNase/RNase-free water was added to Zymo-Spin IIC and IIIC columns to extract RNA and DNA, respectively.

### Gene expression analysis by real-time qPCR

RNA integrity was checked by 1% agarose gel electrophoresis. Concentrations were measured by using a Nanodrop 1000 system spectrophotometer (Thermo Fisher Scientific, Waltham, MA, USA). RNA samples with 260/280 nm absorbance ratio > 1.8 and < 2.0 were subsequently reverse-transcribed with the GeneAmp RNA PCR kit (Life Technologies, Monza, Italy). Relative abundance of each mRNA of interest was assessed by real-time qRT-PCR using the Sybr Green gene expression Master Mix (Life in a Step One Real-Time PCR System, Life Technologies, Monza, Italy) as previously described^51^. Real-time qRT-PCR was applied to quantify the relative expression of the nuclear receptor subfamily 3, group C, member 1 (*Nr3c1*) and member 2 (*Nr3c2*) genes, which code for GR and MR, respectively. All data were normalized to glyceraldehyde-3-phosphate dehydrogenase (*Gapdh*) as the endogenous reference gene. Relative expression of different gene transcripts was calculated by the Delta-Delta Ct (ΔΔCt) method and converted to relative expression ratio (2^−ΔΔCt^) for statistical analysis^52^. The MR/GR balance was calculated as the ratio between the 2^−ΔΔCt^ value for MR and that for GR for each mouse^53^. Primers used for PCR amplification were designed using Primer3web software (version 4.1.0, https://primer3.ut.ee/) and are reported in Table S1. Raw data of the PCR analyses are provided as Supplementary Material.

### DNA methylation analysis

DNA methylation analysis was performed at the Centre for Applied Biomedical Research (CRBA), Sant’Orsola Hospital, Bologna (Italy). DNA extracted from frozen hippocampi (100 ng/µl) was treated with the EZ DNA Methylation Kit (cat. # D5001, Zymo Research, Orange, CA, USA). Bisulfite treatment produced methylation-dependent conversion of cytosine to uracil residues that were differently cleaved by uracil-specific cleavage and, finally, detected by Sequenom MALDI-TOF mass spectrometry. This analysis produced signal pattern pairs indicating non-methylated and methylated DNA, which were then expressed as a ratio. Depending on the sequence of the target region and the distribution of CpGs (–C–phosphate–G–), the mass spectrum could contain multiple signal pairs of cleavage products. DNA methylation levels were assessed for the MR and GR genes using 3 and 5 amplicons, respectively (cf. Table S2 for primers pairs). In line with the exploratory nature of the present study, we analyzed and compared between experimental groups the mean DNA methylation value of each gene. The only exception concerned the nerve growth factor-inducible protein A (NGFI-A) binding region on exon 1_7_ of the GR promoter, a site that was previously and specifically linked to rat maternal behavior^17,54^. Here, we tested whether the hypermethylation of this specific site also occurred in our model. Raw data of methylation are provided as Supplementary Material.

### Statistics

Data were first tested for normal distribution using the Shapiro–Wilk normality test. In case the normality assumption was rejected by the Shapiro–Wilk test, we analyzed data with the Kruskal–Wallis test and applied Dunn’s correction for the post-hoc analyses. In case the Shapiro–Wilk test did not reject the assumption of distribution normality, we performed one- or two-way ANOVA with perinatal treatment as group factor (3 levels). In the two-way repeated-measure ANOVAs performed to analyze the sleep phenotype, we also included the wake-sleep state or the photoperiod transition (4-h bin) or the time of the day as factors. When the ANOVAs showed significant (*p* < 0.05) main effects or interactions, we performed multiple pairwise comparison tests between the 3 experimental groups applying the Tukey’s HSD correction. Pearson’s correlation coefficients were calculated between the amount of wakefulness at LtoD and GR or MR expression data. Variables included in these correlations were also tested for the presence of outliers using the ROUT method (Q value set at 1%) by Prism 7.0 (GraphPad, www.graphpad.com). Data were analyzed with PASW Statistics 18 (SPSS, www.spss.com) or Prism 7.0 (GRAPHPAD, www.graphpad.com) and are reported as mean ± SEM. Degrees of freedom and F (for ANOVAs) or t (for post-hoc tests) or H (for Kruskal–Wallis test) values are reported in the text for main results. Details on the statistical analyses employed are reported in the Statistical Appendix.

## Results

### Baseline recordings

**Figure 2:**
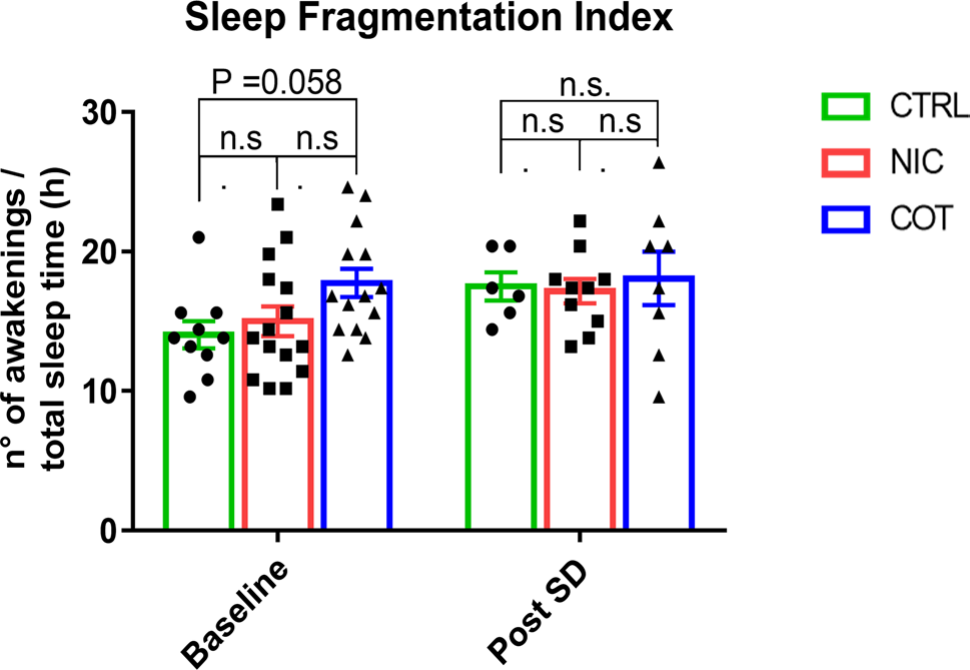
Sleep fragmentation index. Values of sleep fragmentation index calculated as the n° of awakenings/total sleep time (in hours) during 48 h of baseline recordings and 18 h of recordings during recovery after sleep deprivation (SD) by gentle handling for 6 h. Values are shown for adult male mice perinatally exposed to nicotine (NIC, *n* = 15), cotinine (COT, *n* = 14) or just the vehicle (CTRL, *n* = 10). Values after sleep deprivation were calculated on 10 NIC, 8 COT and 6 CTRL mice. n.s., not significant.

Perinatal exposure to either nicotine or cotinine did not impact on the total W, NREMS or REMS time exhibited by adult mice during baseline recordings (Table 1). Also, the number and the duration of the wake-sleep episodes (Table 1) did not significantly differ among experimental groups. The one-way ANOVA indicated that the sleep fragmentation index (Fig. 2) significantly differed among experimental groups (F(2,36) = 3.294, *P* = 0.0486) being higher, but failing to reach the statistical significance, in the COT than in CTRL mice (t(36) = 3.361, *P* = 0.0581).

**Table 1:** Wake-sleep cycle architecture in mice perinatally exposed to nicotine, cotinine or vehicle.

Behavioral state	Measure	CTRL (*n* = 10)	NIC (*n* = 15)	COT (*n* = 14)
Wakefulness	% in 24 h	48 ± 1	49 ± 1	49 ± 1
	No of bouts in 24 h	164 ± 10	172 ± 10	201 ± 13
	Bout duration (sec)	251 ± 18	244 ± 15	215 ± 17
NREMS	% in 24 h	42 ± 2	42 ± 1	40 ± 2
	No of bouts in 24 h	458 ± 11	510 ± 25	500 ± 25
	Bout duration (sec)	79 ± 3	72 ± 4	70 ± 4
REMS	% in 24 h	7 ± 1	6 ± 1	7 ± 1
	No of bouts in 24 h	134 ± 15	135 ± 12	151 ± 23
	Bout duration (sec)	40 ± 2	38 ± 2	40 ± 2

The table shows the total duration (percentage of recording time), the number of bouts and the mean bout duration of wakefulness, non-rapid-eye-movement sleep (NREMS) and rapid-eye-movement sleep (REMS) of adult male mice perinatally exposed to nicotine (NIC), cotinine (COT) or just the vehicle (CTRL) recorded during baseline conditions.

**Figure 3:**
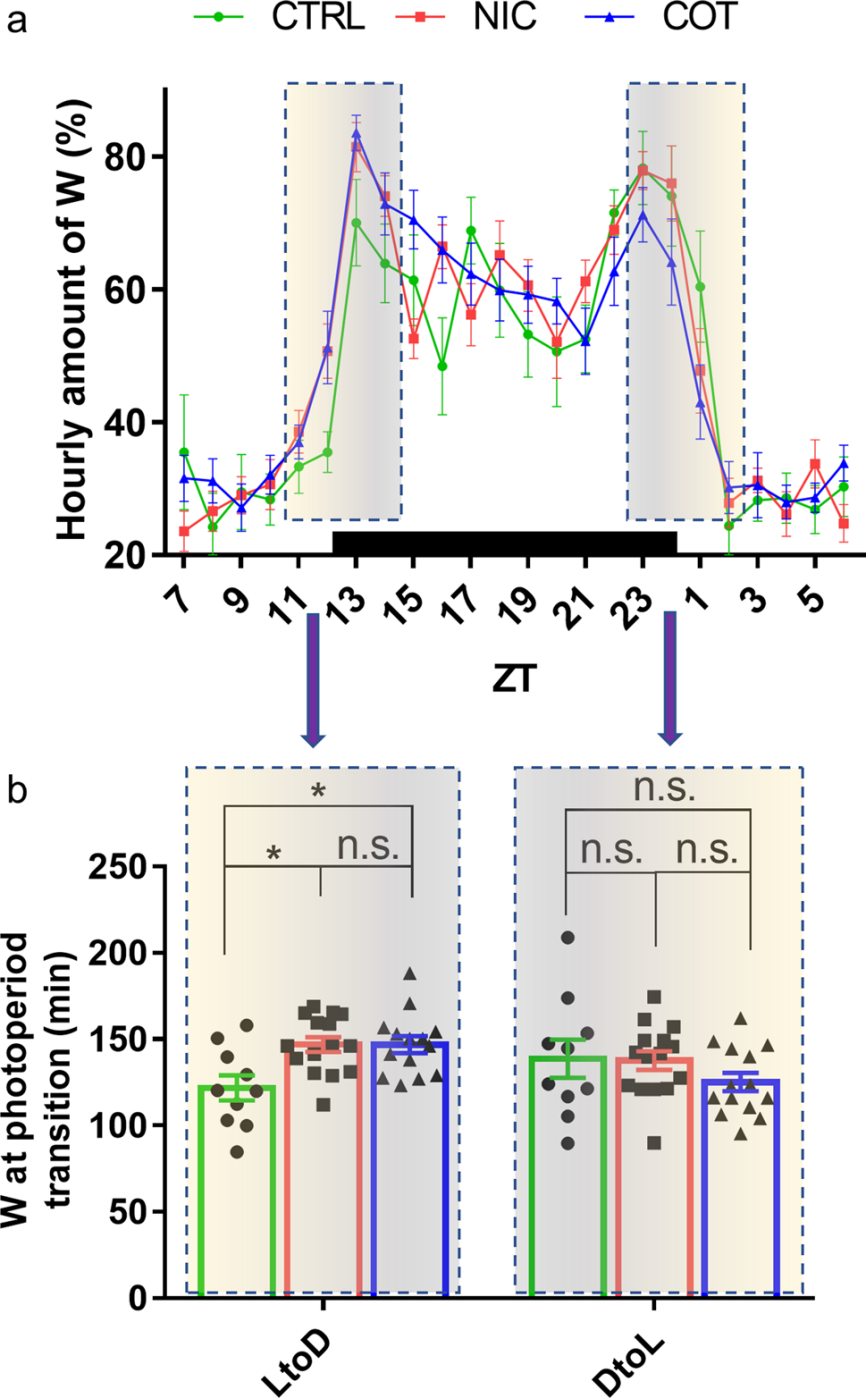
Day-night hourly profiles of the time spent in wakefulness during baseline recordings. Panel (**a**) shows the 24-h hourly profile of the time spent in wakefulness (W) during baseline recordings for adult male mice perinatally exposed to nicotine (NIC, *n* = 15), cotinine (COT, *n* = 14) or just the vehicle (CTRL, *n* = 10). Mice were left undisturbed with lights on at 9.00 a.m. (Zeitgeber Time 0, ZT0) and lights off at 9.00 p.m. (ZT12). Panel (**b**) shows the amount of W in the 4 h surrounding the photoperiod transitions. In particular, values for light to dark (LtoD, ZT10–ZT14) and dark to light (DtoL, ZT22–ZT2) transitions are shown. *p (adjusted for multiple comparisons) < 0.05; n.s., not significant.

The 24-h profiles of the wake-sleep states (calculated as mean values of 2 consecutive 24-h recordings) were largely overlapping between the 3 experimental groups (see wakefulness profile in Fig. 3a and examples of raw data in Fig. S1). However, there was a statistically significant interaction between the perinatal drug treatment and the photoperiod transition (F(2,36) = 3.576, *P* = 0.0383) on the amount of wakefulness during the 4 h surrounding the photoperiod transitions (Fig. 3b). Post-hoc analyses revealed that both NIC and COT mice spent significantly more time awake at the LtoD transition than CTRL mice (t(72) = 3.931, *P* = 0.0188, and t(72) = 3.869, *P* = 0.0211, respectively) whereas no significant difference between groups was found at the DtoL transition (Fig. 3b).

**Figure 4:**
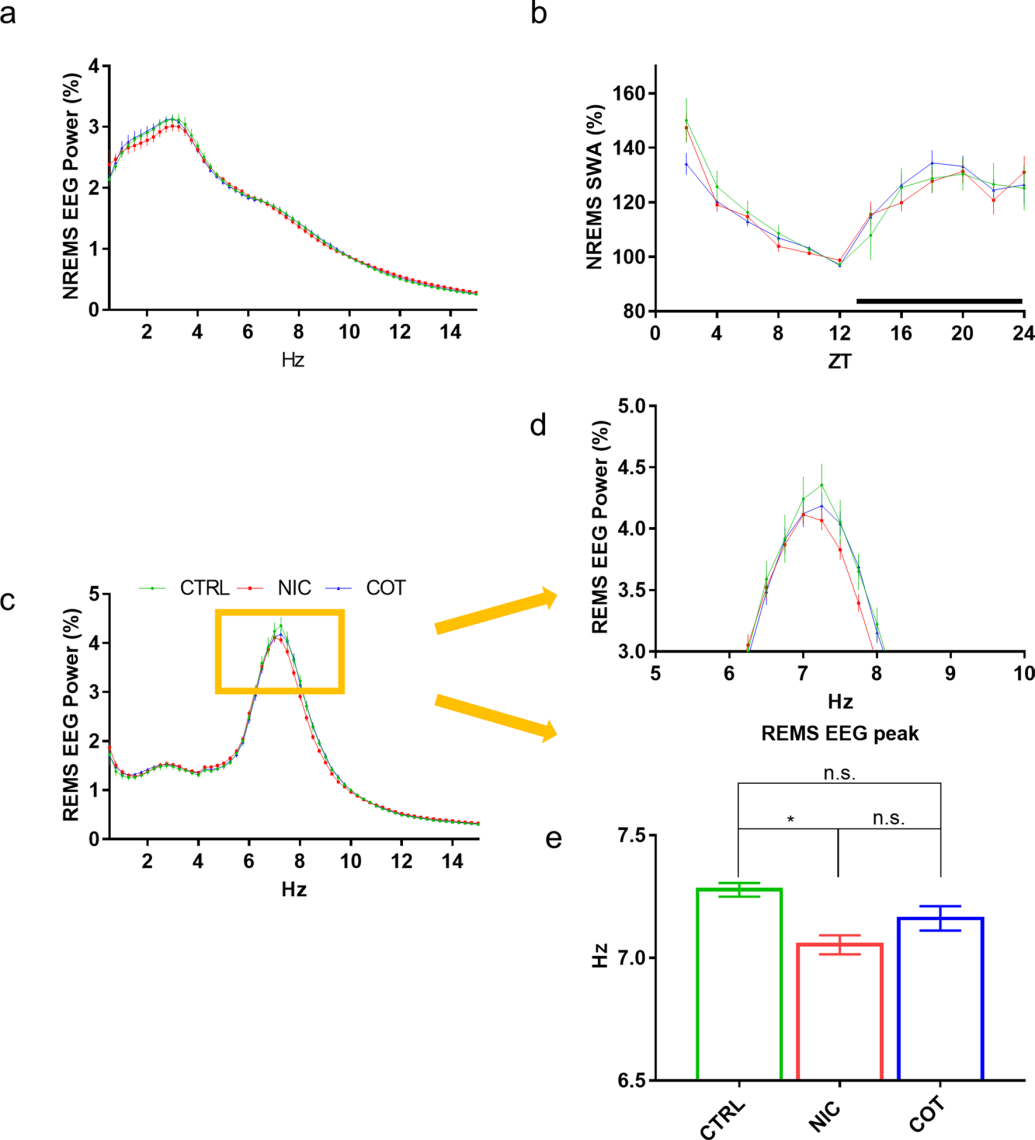
Electroencephalogram analysis in sleep during baseline recordings. Panel (**a**) shows electroencephalographic (EEG) power spectral density in baseline recordings during non-rapid-eye-movement sleep (NREMS) expressed as a percentage of total EEG spectral power. Panel (**b**) shows power in the delta frequency range (1–4 Hz, EEG slow-wave activity, SWA) during NREMS in baseline conditions. EEG SWA was normalized to values in the last 4 h of the light period. Panel (**c**) shows EEG power spectral density in baseline recordings during rapid-eye-movement sleep (REMS) expressed as a percentage of total EEG spectral power. Panel (**d**) shows a magnification of the orange square in panel (**c**). Panel (**e**) shows the values of the EEG peak during REMS. All data refer to adult male mice perinatally exposed to nicotine (NIC, *n* = 14), cotinine (COT, *n* = 14) or just the vehicle (CTRL, *n* = 11). *p (adjusted for multiple comparisons) < 0.05; n.s., not significant.

NIC and COT mice did not show any significant difference with respect to CTRL mice in the EEG power spectra or in the SWA values during NREMS (Fig. 4a, b). On the other hand, the EEG power spectrum peak during REMS differed among groups (H(37) = 10.91, *P* = 0.0043) occurring at a significantly lower frequency in NIC mice than in CTRL mice (*P* = 0.0032, Fig. 4c-e).

### Sleep deprivation and recovery

After baseline recordings, we sleep deprived mice by gentle handling for 6 h (from ZT0 to ZT6) and then we recorded their sleep rebound for the following 18 h. Sleep deprivation was effective as indicated by the almost complete absence of sleep during the 6 h of this protocol (Fig. S2), and by the rapid and wide increase in SWA during sleep recovery after deprivation (Fig. S3b) compared with baseline recordings at the same ZT (Fig. 4b). During the sleep recovery period, we found no significant difference among experimental groups in the % of time spent in wakefulness, NREMS or REMS (Table S3). Even though the sleep patterns exhibited after sleep deprivation by the 3 groups were similar to those during the baseline recordings, we failed to find any statistically significant difference among experimental groups in terms of sleep fragmentation index (Fig. 2), time spent awake at photoperiod transition (Fig. S2), EEG power spectra, and SWA (Fig. S3b) during recovery after sleep deprivation.

### Gene expression analysis

**Figure 5:**
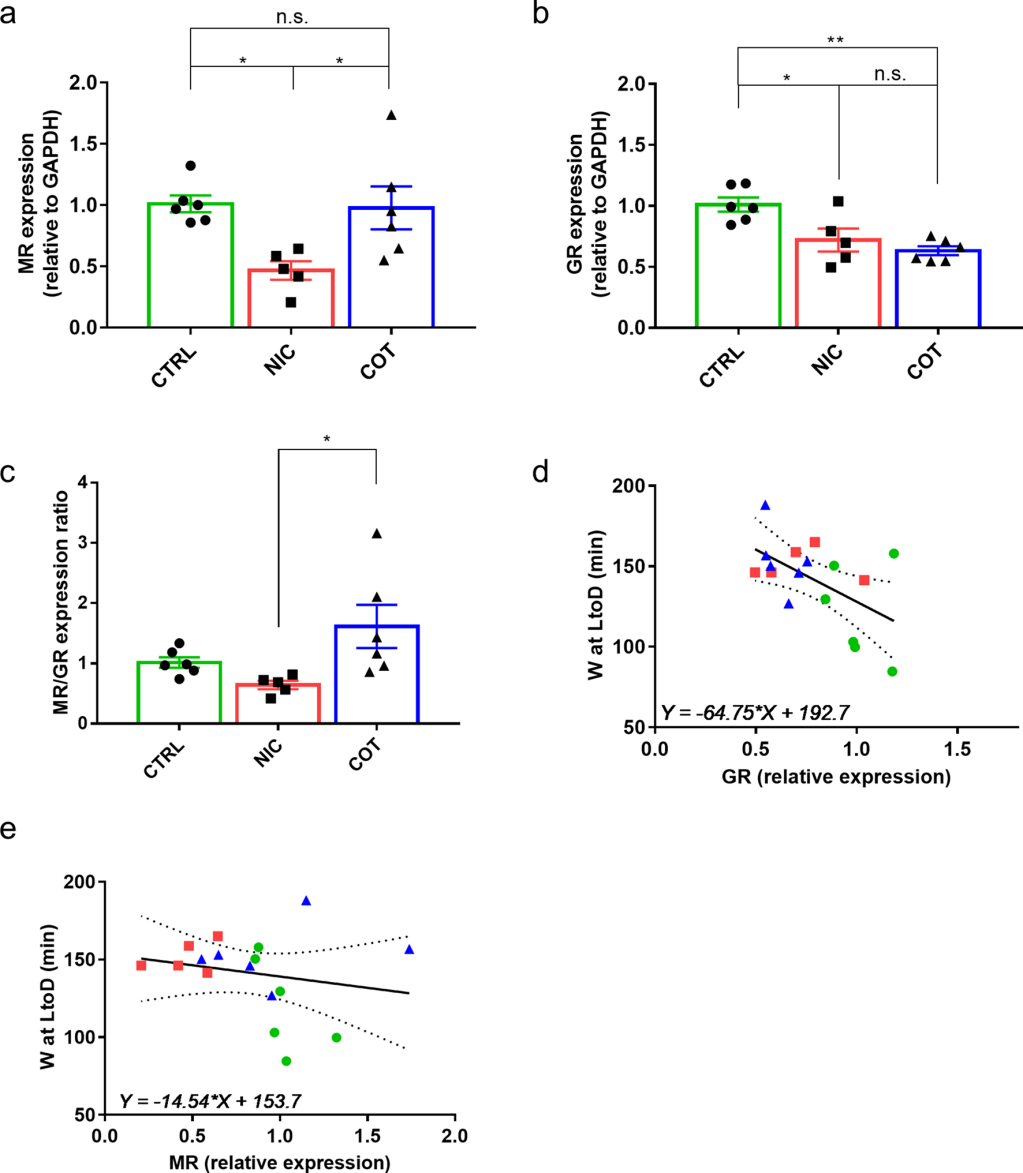
Corticosteroid receptor gene expression in hippocampus. Panel (**a**, **b**) show the expression of hippocampal GR (Nr3c1) and MR (Nr3c2) genes relative to the expression of glyceraldehyde-3-phosphate dehydrogenase (Gapdh), used as reference gene. Panel (**c**) shows the expression ratio of MR and GR genes. Panels (**d**, **e**) show the correlation between the relative hippocampal GR (panel **d**) or MR (panel **e**) expression (x-axis) and the amount of wakefulness (W) at light to dark (LtoD) transition (y-axis) considering all the mice of the present experiment. Continuous and dotted lines indicate the regression line and the 95% confidence, respectively. All data refer to adult male mice perinatally exposed to nicotine (NIC, *n* = 5), cotinine (COT, *n* = 6) or just the vehicle (CTRL, *n* = 6). *p (adjusted for multiple comparisons) < 0.05; **p (adjusted for multiple comparisons) < 0.01; n.s., not significant.

The hippocampal expression of both GR and MR was significantly affected by perinatal drug exposure (F(2,14) = 10.22, *P* = 0.0018, and F(2,14) = 5.853, *P* = 0.0142, respectively). The expression of both GR and MR was downregulated in NIC compared to CTRL mice (t(14) = 4.506, *P* = 0.0170 and t(14) = 4.379, *P* = 0.0202, respectively). The expression of GR (t(14) = 6.134, *P* = 0.0018) but not that of MR (t(14) = 0.2772, *P* = 0.9791) was downregulated in COT compared to CTRL mice (Fig. 5a, b). The expression ratio of the 2 hippocampal corticosteroid receptors (MR/GR) significantly differed between groups (F(2,14) = 4.473, *P* = 0.0315) being significantly lower in NIC mice than in COT mice (t(14) = 4.154, *P* = 0.0274, Fig. 5c). However, neither NIC mice nor COT mice showed significant differences in the MR/GR expression ratio compared to CTRL mice. Finally, we tested the correlation between either GR or MR expression and the amount of time spent awake at LtoD transition (Fig. 5d, e). These correlations were computed on the whole mouse dataset since the ROUT method did not show any outlier in any of the variables considered. We found that GR expression significantly and inversely correlated with the amount of wakefulness at LtoD transition (r^2^ = 0.3079, *P* = 0.0208, Fig. 5d) while no significant correlation was found when MR expression was considered (r^2^ = 0.0146, *P* = 0.647, Fig. 5e).

### DNA methylation analysis

**Figure 6:**
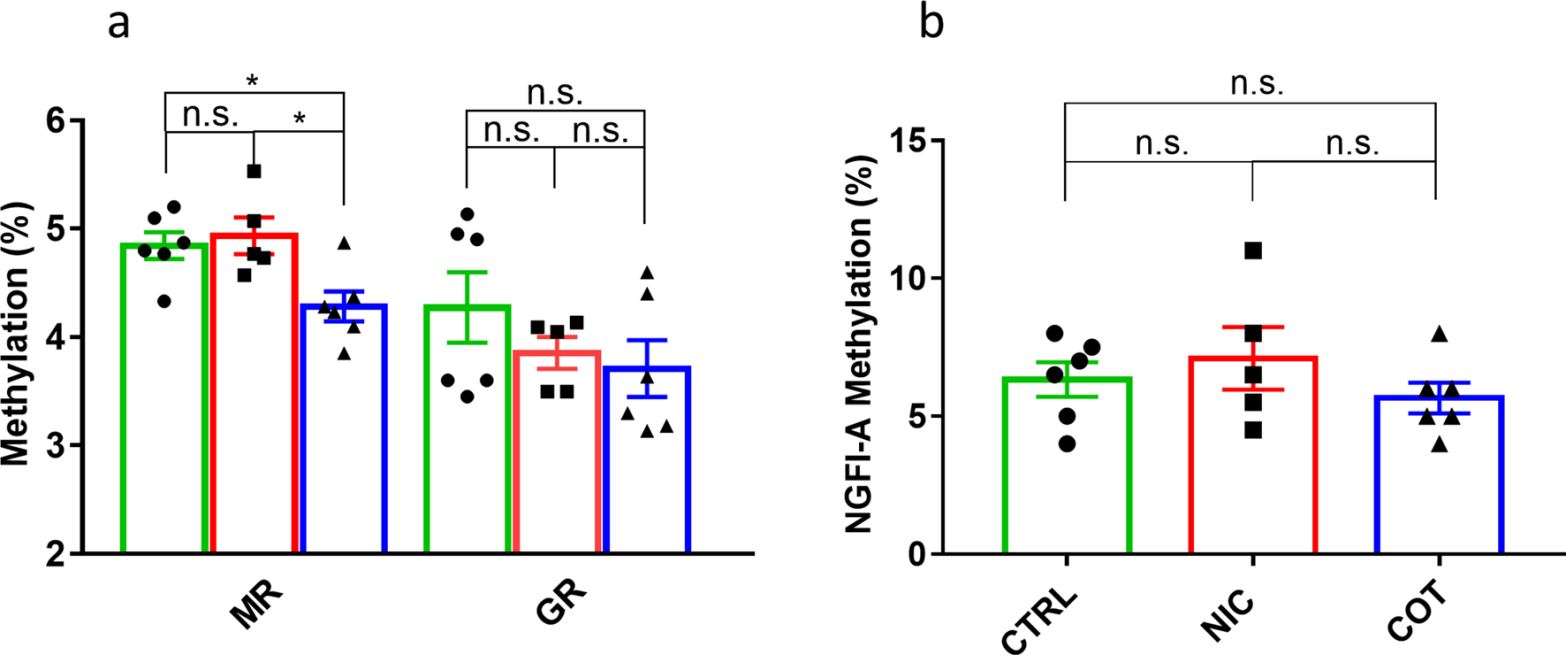
DNA methylation analysis of hippocampal corticosteroid receptors. Panel (**a**) shows the mean % DNA methylation status of hippocampal GR (Nr3c1) and MR (Nr3c2) genes. Panel (**b**) shows the mean % DNA methylation status of the NFGI-A binding site, a specific sequence present on the GR gene. All data refer to adult male mice perinatally exposed to nicotine (NIC, *n* = 5), cotinine (COT, *n* = 6) or just the vehicle (CTRL, *n* = 6). *p (adjusted for multiple comparisons) < 0.05; n.s., not significant.

The global methylation status of the promoter regions of GR (Nr3c1) and MR (Nr3c2) genes was assessed in the hippocampus (Fig. 6a). No difference was found in the mean methylation pattern of the GR promoter among experimental groups (F(2,14) = 1.27, *P* = 0.3113) whereas we found a significant main effect of the perinatal drug treatment on MR methylation (F(2,14) = 10.8, *P* = 0.0015. Post-hoc analysis indicated that significant mean hypomethylation was present in COT but not in NIC mice compared to CTRL mice (t(14) = 5.149, *P* = 0.0071, and t(14) = 1.135, *P* = 0.7077, respectively). Moreover, COT mice showed significant hypomethylation also compared to NIC mice (t(14) = 6.044, *P* = 0.0021). Then, we specifically analyzed the percentage of methylation of the NGFI-A binding region present on the exon 1_7_ of the GR promoter, which was previously linked to rat maternal behavior^17,54^. In the present experiment, however, perinatal drug exposure did not significantly alter the specific methylation level of the NGFI-A binding region in the GR promoter (F(2,14) = 0.837, *P* = 0.4536, Fig. 6b).

## Discussion

The present study produced the following main results: (a) the perinatal exposure to either nicotine or to its principal metabolite, cotinine, entailed a long-lasting reprogramming of the percent of time awake at the light-to-dark transition in mice; (b) early-life exposure to nicotine or cotinine modified the expression and the balance of hippocampal corticosteroid receptors; (c) the hippocampal expression of GR inversely correlated with the percent of time awake at the light-to-dark transition.

To our knowledge, only one previous experiment investigated the effect of perinatal nicotine exposure on sleep phenotype of adult mice^33^, showing that these mice spent more time in NREMS during the inactive (light) phase, and, after 5 h of sleep-deprivation, slept more than controls^33^. Our results are in contrast with these findings. In NIC mice, we found a significant increase of time spent awake at LtoD transition (Fig. 3b), with no significant change in sleep recovery after sleep deprivation (Fig. S2 and S3). At least in part, this might be due to that plasma cotinine concentration in the dams was approximately three times as high in our study^7^ as in previous work^33^. Unfortunately, it is not possible to directly compare the level of indirect exposure between our pups and those in that previous study, in which these data were not provided^33^. However, we were able to replicate the main results on sleep alterations and GR expression downregulation in independent experimental groups of mice (NIC and COT), in which plasma cotinine levels were similar^7^.

Another interesting finding of our study was that the EEG power spectrum peak recorded during REMS in NIC mice, but not that in COT mice, occurred at slightly but significantly lower frequencies than that of the CTRL group. In rodents, the predominant EEG rhythm during REMS is in the theta frequency, which mainly represents the pattern of discharge of hippocampal neurons^55^. Hippocampal theta activity is influenced by circulating cortisol (corticosterone) through the activation of corticosteroid receptors^56^. It has been demonstrated that injection of specific MR antagonists significantly reduces the theta peak amplitude^56^. Intriguingly, we found a slowing of the EEG theta frequency during REMS in NIC mice, which were the only group with a concomitant hippocampal MR downregulation (Fig. 5a). Our data thus support a role of hippocampal MR in the modulation of the EEG theta rhythm during REMS.

Even though the sleep patterns of the 3 experimental groups after 6 h of sleep deprivation broadly agreed with those of the baseline recordings, higher variability associated with the sleep homeostasis challenge obscured the statistical differences we described with the mice left undisturbed (Figs. 2, 3 and 4 and Fig. S2-S3). This negative result cannot be attributed to incomplete sleep deprivation, considering the almost total absence of sleep during the 6 h of this protocol (Fig. S2) followed by a large increase in the SWA values (Fig S3b) recorded immediately upon recovery. On the one hand, this result indicates that the increase in wakefulness at the LtoD transition during baseline conditions in the NIC and COT groups was not critically related to defects in sleep homeostasis. It is tempting to speculate that this sleep alteration was obscured during recovery after sleep deprivation by the heightened level of stress induced in all study groups by the gentle handling deprivation procedure^57^.

In the second part of the present study, we focused on the molecular analysis of hippocampal corticosteroid receptors both at RNA and DNA level. We found that perinatal nicotine exposure was associated with downregulation of the expression of both GR and MR in the hippocampus (Fig. 5a, b), which is a condition associated with HPA axis hyper-activity and consequent increase of circulating cortisol (corticosterone) levels^22,23^. COT mice showed a significant downregulation of hippocampal GR, but not of MR, compared to CTRL mice. Nevertheless, significant alterations in the hippocampal MR/GR expression ratio (Fig. 5c), which may have been associated with stress-related brain disorders^20,21^ and altered stress response homeostasis^14^, occurred between NIC and COT mice, but not between either of these groups and the CTRL group of mice. This result should be viewed with caution, as it may reflect a false positivity of the difference between NIC and COT or a false negativity of the difference between CTRL and either NIC or COT. Nevertheless, this finding does suggest that, at least in terms of the MR/GR expression ratio, nicotine and cotinine exposures may produce significant contrasting effects. This would be in line with the hypothesis that cotinine should not be considered just as an inert byproduct of nicotine metabolism^30^. The physiological roles of hippocampal MR are still debated. However, MR is relevant to alterations in memory consolidation and cognitive task performance, to psychological alterations, and to depression^58,59^. Interestingly, all these alterations have already been linked to perinatal exposure to nicotine and to hippocampal dysfunction^60,61^.

On the same hippocampal samples, we analyzed the DNA methylation pattern of the promoter regions of GR (*Nr3c1*) and MR (*Nr3c2*) (Fig. 6a, b). The methylation status of the GR promoter and specifically of the NGFI-A binding site as a function of perinatal stress has been widely investigated in different species^5^. Most, but not all studies in rodents showed increased DNA methylation of the GR promoter associated with various psychological perinatal stressors, including impaired maternal care or maternal separation^5,17,54^. On this basis, we tested DNA methylation of the GR promoter considering nicotine or cotinine as pharmacological perinatal stressors. We conducted an extensive analysis of the methylation status of the GR promoter region including 181 CpGs and using 5 pairs of amplicons. We also included specific sites already proved to be linked with perinatal stress exposure^5,17,54,62^ and we ran the same analysis twice. Nonetheless, we failed to find any significant change of the methylation status in the GR promoter region (Fig. 6a) and in the NGFI-A binding site (Fig. 6b) of either NIC or COT mice, which could be related to the receptor downregulation. These data may suggest that the methylation of the GR promoter and of the NGFI-A binding site are more related to psychological rather than to pharmacological perinatal stressors. To our knowledge, evidence on the hippocampal MR methylation status was provided in mice by one study, in which MR hypomethylation in the offspring was related to the administration of glucocorticoids to their fathers before mating^62^. Our data showed DNA hypomethylation of the same MR region only in the COT group mice (Fig. 6a). However, this molecular finding was not associated with differential MR expression (Fig. 6b) thus suggesting the occurrence of other counterbalancing epigenetic modifications elsewhere in the same gene.

Concomitant with the investigation of the long-term consequences of perinatal nicotine exposure, our experimental protocol allowed to test whether the same effects could be elicited by the perinatal exposure to cotinine. We found that the sleep phenotype and the corticosteroid receptor gene expression and methylation pattern of COT mice broadly, but not entirely, overlapped those of the NIC group. Most, but not all, of the changes we described in response to nicotine may not have been directly elicited by nicotine exposure, but, rather by cotinine exposure. While this hypothesis may well be correct only for selected biomarkers, it would appear in line with the available evidence that suggests considering cotinine a biologically active and potentially harmful molecule^30^. On the other hand, we found evidence of diverging actions of perinatal nicotine vs. cotinine exposure on MR methylation (Fig. 6a) and expression (Fig. 5a, c). These differences may depend on partially diverging pharmacodynamics and/or pharmacokinetics of nicotine vs. cotinine. Cotinine is a positive allosteric modulator of nicotine receptors^30^, has a much longer half-life than nicotine, and can also bind at least 1 different receptors^29^. Finally, it cannot be excluded that nicotine catabolism produces active molecules other than cotinine^26,30^.

The mechanisms underlying the long-term sleep alterations after perinatal exposure to nicotine or cotinine remain unclear. Maternal smoking may impact on the developing brain’s acetylcholine neurotransmitter system or on neurons involved in sleep regulation^33–36^. We raise the hypothesis that maternal smoking represents a stressor impacting on offspring hippocampal neurodevelopment and, through that, on adult sleep regulation^2^. According to this hypothesis, perinatal exposure to either nicotine or cotinine would represent a form of early-life stress producing fetus overexposure to steroids^5,17,54^, would alter the hippocampal expression of corticosteroid receptors. This modulation would then dampen the inhibitory role of the hippocampus on the HPA axis activity. Persistent hyperactivation of the HPA axis would then cause persistent alterations of the sleep phenotype of early-life stressed subjects^63,64^. Accordingly, we demonstrated the concomitant presence of sleep alterations (Figs. 3, 4) and downregulation of hippocampal corticosteroid receptors (Fig. 5) in adult mice perinatally exposed to nicotine. In line with this theory, moreover, we found that NIC mice stayed awake longer than CTRL group at the LtoD transition, which corresponds to the human cortisol awakening response^50^. This theory should be regarded as a working hypothesis, with many critical predictions that still need to be tested. Importantly, the theory predicts an increase in circulating corticosterone in NIC and COT with respect to CTRL mice at the LtoD transition in baseline conditions, but not during recovery after sleep deprivation, which may increase corticosterone secretion also in CTRL mice^57^. This hypothesis should be tested in future studies, as our present experiments did not include corticosterone measurement. On the other hand, our failure to detect epigenetic changes in methylation associated with corticosteroid receptor downregulation in NIC and COT mice was not sufficient to rule out a role of the epigenetic inheritance system. Further studies should investigate other types of modifications (e.g. histone modification, non-coding RNAs)^2^ that may operate outside the specific promoter regions/genes we looked at.

Some limitations of the present study must be acknowledged. The results of the present study might be, at least in part, due to indirect effects of nicotine and/or cotinine exposure on maternal drinking behavior^42^ and pup care^65^ rather than to direct effects on the HPA axis development. Both these aspects, not included in our experiment, should be further investigated. Another limitation of the present study is that we analyzed the hippocampus as a whole, without evaluating the presence of regional differences in the corticosteroid receptor expression. Moreover, due to technical limitations in sample purification, we could not quantify the protein levels of corticosteroid receptors in the same samples in which we investigated the DNA methylation and RNA expression. Caution must be exercised in interpreting correlations between hippocampal MR/GR expression and sleep phenotype. Significant correlations do not directly imply causal links. Moreover, although we took care to ensure that no outlier data were included in the correlation analyses, our results cannot be considered robust (as they relied on a relatively small number of data points) and should be considered as preliminary until replicated on larger samples. An important limitation of our study is that we did not quantify plasma corticosterone levels. Future specific experiments should be performed to address this point. Finally, we did not investigate sex differences in the long-term effects of the exposure to nicotine and/or cotinine. Others have shown that perinatal nicotine exposure differentially impacts on adult sleep in males vs. females^33^, and suggested that sex differences in receptors for the stress hormones, such as the hippocampal GR, could make females more susceptible to stress system dysregulation after a stressful event^66^. On this basis, we hypothesize that our protocol would produce different and more evident alterations in adult females than in adult males.

In conclusion, we found evidence that perinatal nicotine and cotinine exposure produced long-lasting alterations in adult mice on two correlated aspects: the wake-sleep phenotype and the hippocampal expression of corticosteroid receptors. These data support the view that our adult sleep phenotype, and its potential alterations, can be associated with early-life events during pregnancy and lactation.

## Supplementary Information

Supplementary Information 1.

Supplementary Information 2.

Supplementary Information 3.

Supplementary Information 4.

Supplementary Information 5.

Supplementary Information 6.

Supplementary Information 7.

## Data Availability

Raw data of the present experiment are available upon reasonable request.
