# Supplementary material for: Early-life nicotine or cotinine exposure produces long-lasting sleep alterations and downregulation of hippocampal corticosteroid receptors in adult mice
Source: Sci Rep. 2021 Dec 13;11:23897. doi: 10.1038/s41598-021-03468-5 (PMC8668915; doi:10.1038/s41598-021-03468-5)
Supplement: Supplementary file 2 — Supplementary Information 2. [file 41598_2021_3468_MOESM2_ESM.docx]

**Table S1. Primer sequences used for real-time qPCR.**

| **Gene** | **Accession Number** | **Sense Primer (5’ – 3’)** | **Antisense Primer (5’ – 3’)** |
| --- | --- | --- | --- |
| **Glyceraldehyde-3-phosphate dehydrogenase (Gapdh)** | NM_008084 | CGTGCCGCCTGGAGAAACC | TGGAAGAGTGGGAGTTGCTGTTG |
| **Glucocorticoid receptor (Nr3c1)** | DQ504162 | AATGAGACCAGATGTGAGTTC | GGTAATTGTGCTGTCCTTCC |
| **Mineralocorticoid receptor (Nr3c2)** | [NM_001083906.2](https://www.ncbi.nlm.nih.gov/entrez/viewer.fcgi?db=nucleotide&id=1402371341) | GAAGAGCCCCTCTGTTTGCAG | TCCTTGAGTGATGGACTGTG |

**Table S2. Primer sequences used for DNA methylation analysis.**

| Gene | Accession Number | Amplicon | **Sense Primer (5’ – 3’)** | **Antisense Primer (5’ – 3’)** |
| --- | --- | --- | --- | --- |
| **Glucocorticoid receptor (Nr3c1)** | NC_000084 | 1 | TTATGAATTTGGTTGATTTGGTTTT | TCCTCTACCTAACCTCTTAAAAATCTC |
|  |  | 2 | GGTATTTGTGGGGGAGTAAAAGTTA | AACCAACATCCAAACTCCCC |
|  |  | 3 | GAGGTATTGAGTTTGGAGTAGTAAATG | AAATTACAAAACAAAACCCACCC |
|  |  | 4 | GGTGGGTTTTGTTTTGTAATTTTTT | AATTTCTTTAATTTCTCTTCTCCCTAA |
|  |  | 5 | TTTTTTTATGGAAAAGAGGGGG | AACTAACAAAAATTTACCAAATCCC |
| **Mineralocorticoid receptor (Nr3c2)** | NC_000074.7 | 1 | GTTATTTTAGTTTTGGGAGGGGG | CAAAATAACAATTACTACACTCACCTTTTC |
|  |  | 2 | GTTGTAGGTGGAGAGAGTAAGGGTT | AACCATTTCCATTAACCAAAAAAAA |
|  |  | 3 | TTTTTGGTTAATGGAAATGGTTTTA | CCTATTACAATACTTACCACCTCCAC |

**Table S3. Wake-sleep architecture after 6 hours of sleep deprivation in mice perinatally exposed to nicotine, cotinine or vehicle**.

| **Behavioral State** | **Measure** | **CTRL**  **(n = 6)** | **NIC**  **(n = 10)** | **COT**  **(n = 8)** |
| --- | --- | --- | --- | --- |
| **Wakefulness** | % in 18h | 43 ± 2 | 47 ± 2 | 44 ± 4 |
|  | n° of bouts in 18h | 171 ± 12 | 154 ±6 | 165 ±15 |
|  | Bout duration (sec) | 213 ± 22 | 249 ± 12 | 231 ± 12 |
| **NREMS** | % in 18h | 48 ± 2 | 43 ± 1 | 45 ± 3 |
|  | n° of bouts in 18h | 486 ± 28 | 498 ±22 | 484 ± 15 |
|  | Bout duration (sec) | 85 ± 5 | 76 ± 4 | 85 ± 8 |
| **REMS** | % in 18h | 7 ± 1 | 7 ± 1 | 7 ± 1 |
|  | n° of bouts in 18h | 122 ± 16 | 135 ±13 | 130 ± 8 |
|  | Bout duration (sec) | 44 ± 5 | 44 ± 4 | 44 ± 2 |

The table shows the total duration (percentage of recording time), the number of events and the mean bout duration of wakefulness, non-rapid-eye-movement sleep (NREMS) and rapid-eye-movement sleep (REMS) of adult male mice perinatally exposed to nicotine (NIC), cotinine (COT) or just the vehicle (CTRL) recorded after 6 hours of sleep deprivation.
