# Supplementary material for: Early-life nicotine or cotinine exposure produces long-lasting sleep alterations and downregulation of hippocampal corticosteroid receptors in adult mice
Source: Sci Rep. 2021 Dec 13;11:23897. doi: 10.1038/s41598-021-03468-5 (PMC8668915; doi:10.1038/s41598-021-03468-5)
Supplement: Supplementary file 5 — Supplementary Information 5. [file 41598_2021_3468_MOESM5_ESM.pdf]

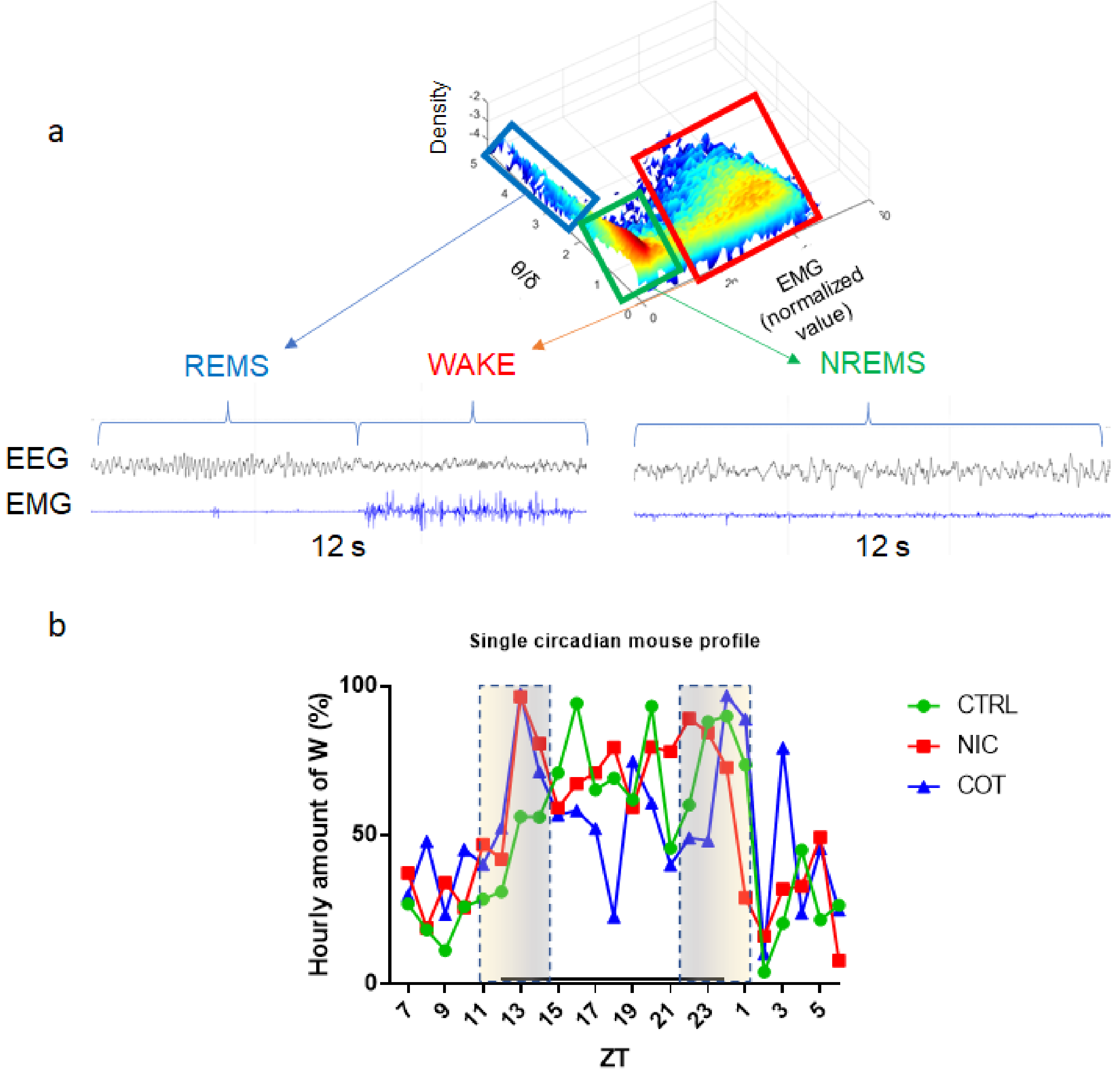

Figure S1. Examples of raw sleep data.

Panel A shows a typical distribution profile of all the 4-s epoch recordings in a single mouse. The distribution is based on electromyogram (EMG) root mean square values (x axis) and on the ratio between θ band (6–9 Hz) and δ band (0.5–4 Hz) spectral power of the electroencephalogram (EEG, y axis). On the z axis, a colorimetric scale represents the fraction of epochs characterized by each given combination of EMG and EEG values. According to our previous publication[45], 3 clusters of epochs can be defined: wakefulness (red), non-rapid-eye-movement sleep (NREMS, green) and rapid-eye-movement sleep (REMS, blue). In the lower part of panel A, examples of raw EEG and EMG recordings during Wakefulness, NREMS or REMS are reported.

Panel B shows the circadian profiles of the amount of time spent awake by 3 representative adult male mice, which were perinatally exposed to nicotine (NIC), cotinine (COT), and just the vehicle (CTRL), respectively. Light-to-dark and dark-to-light transition periods are highlighted by colored rectangles.
