# Supplementary material for: Early-life nicotine or cotinine exposure produces long-lasting sleep alterations and downregulation of hippocampal corticosteroid receptors in adult mice
Source: Sci Rep. 2021 Dec 13;11:23897. doi: 10.1038/s41598-021-03468-5 (PMC8668915; doi:10.1038/s41598-021-03468-5)
Supplement: Supplementary file 6 — Supplementary Information 6. [file 41598_2021_3468_MOESM6_ESM.pdf]

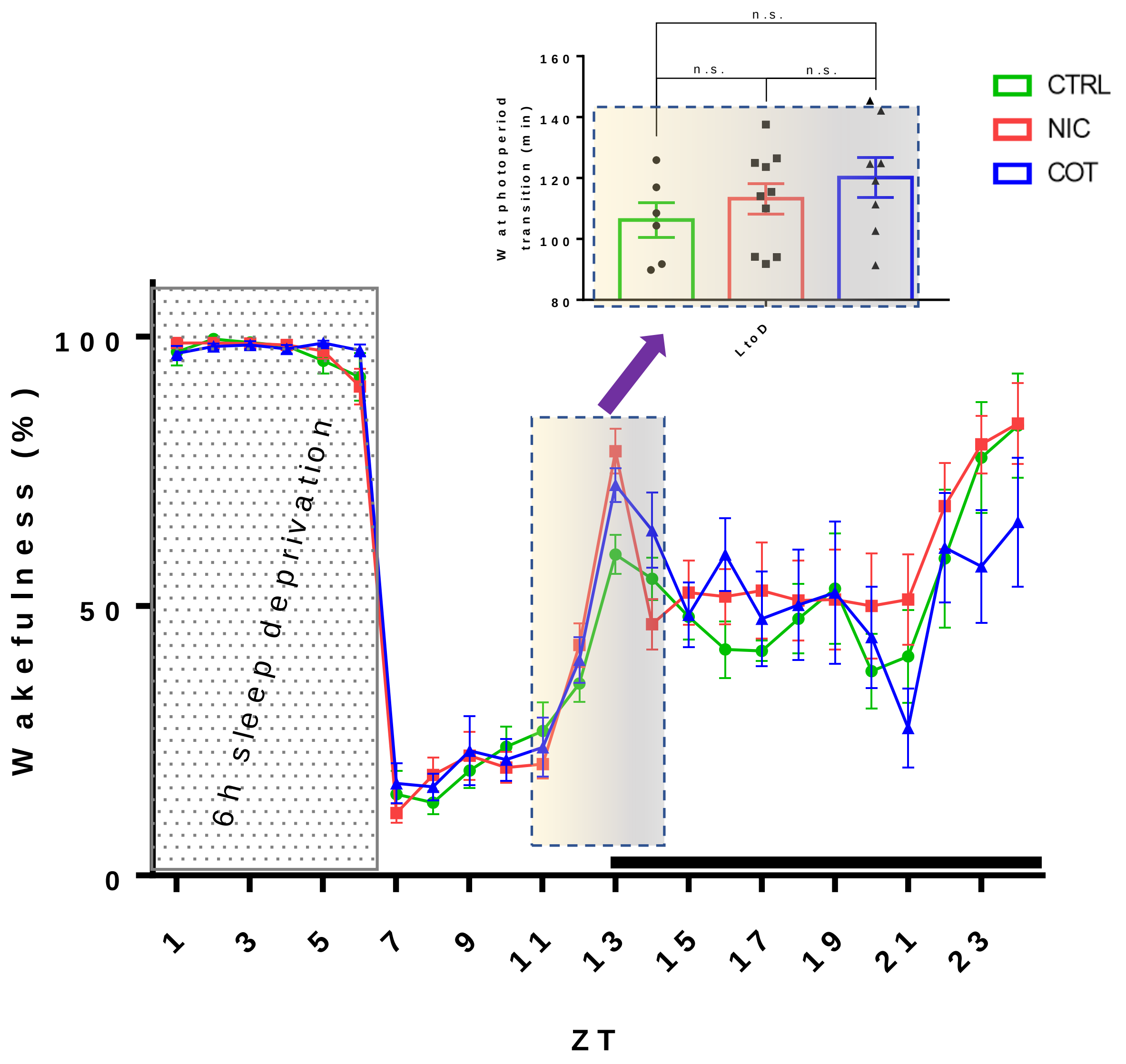

Figure S2. Day-night hourly profiles of the time spent in wakefulness during recovery after sleep deprivation.

Panel A shows the 24-h hourly profile of the time spent in wakefulness (W) during 6 h of sleep deprivation and the following 18 h of recovery in adult male mice perinatally exposed to nicotine (NIC, n = 10), cotinine (COT, n = 8) or just the vehicle (CTRL, n = 6). Sleep deprivation was performed for 6 h by gentle handling from lights on (Zeitgeber Time 0, ZT0) to ZT6. The inset shows the amount of W in the 4 h surrounding the light-to-dark transition (LtoD, ZT10 – ZT14). n.s., not significant.
