# Supplementary material for: Early-life nicotine or cotinine exposure produces long-lasting sleep alterations and downregulation of hippocampal corticosteroid receptors in adult mice
Source: Sci Rep. 2021 Dec 13;11:23897. doi: 10.1038/s41598-021-03468-5 (PMC8668915; doi:10.1038/s41598-021-03468-5)
Supplement: Supplementary file 7 — Supplementary Information 7. [file 41598_2021_3468_MOESM7_ESM.pdf]

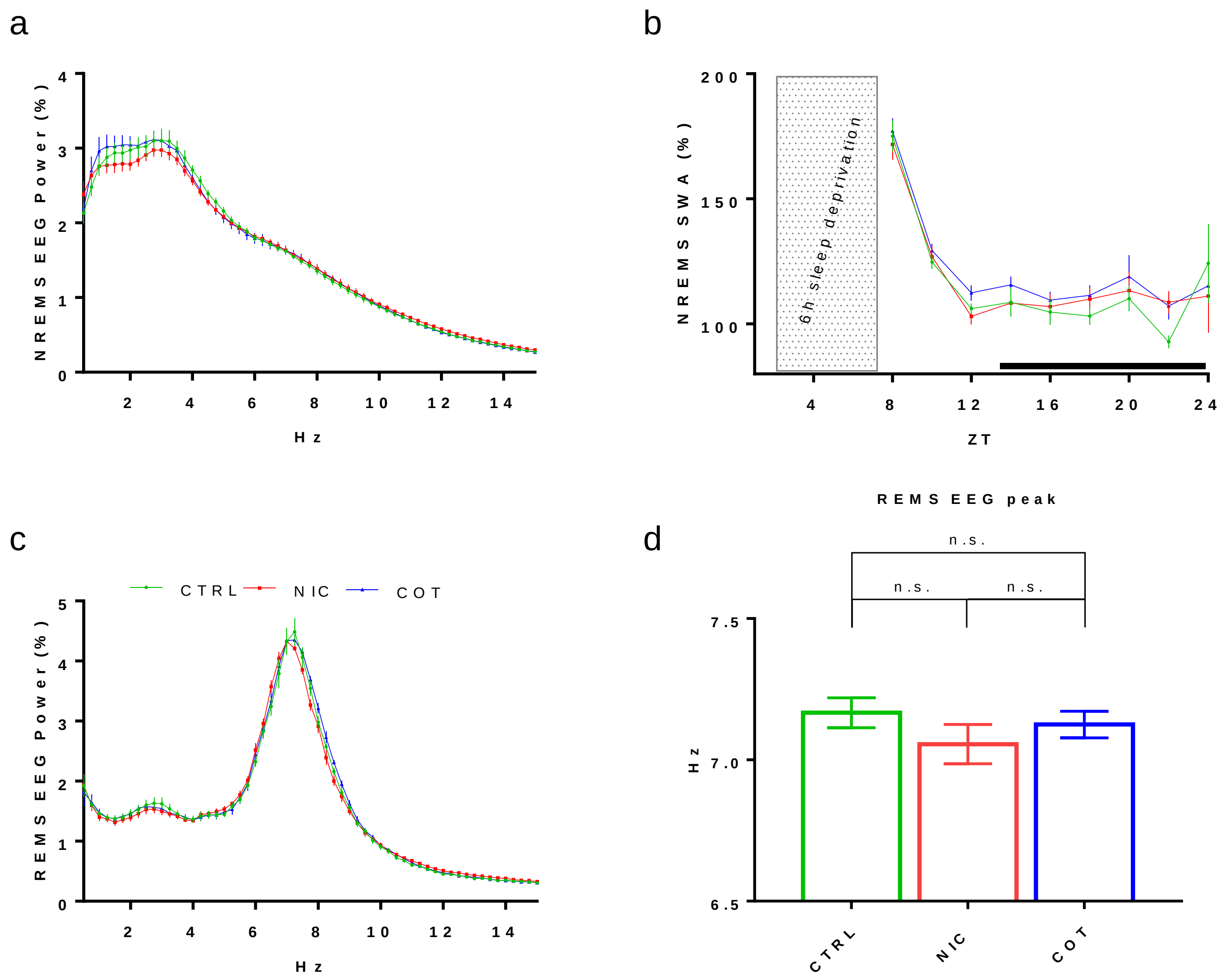

Figure S3. Electroencephalogram analysis after 6 hour of sleep deprivation.

Panel a shows electroencephalographic (EEG) power spectral density during non-rapid-eye-movement sleep (NREMS) after 6 h of sleep deprivation, expressed as a percentage of total EEG spectral power. Panel b shows power in the δ frequency range (1-4 Hz, EEG slow-wave activity, SWA) during NREMS after 6 h of sleep deprivation. EEG SWA was normalized to values in the last 4 hours of the light period. Panel c shows EEG power spectral density during rapid-eye-movement sleep (REMS) after 6 h of sleep deprivation, expressed as a percentage of total EEG spectral power. The inset shows the values of the frequency corresponding to the EEG spectral power peak during REMS.

All data refer to adult male mice perinatally exposed to nicotine (NIC, n = 10), cotinine (COT, n = 8) or just the vehicle (CTRL, n = 6). n.s., not significant.
